# Attention-Based Spatial–Temporal Convolution Gated Recurrent Unit for Traffic Flow Forecasting

**DOI:** 10.3390/e25060938

**Published:** 2023-06-14

**Authors:** Qingyong Zhang, Wanfeng Chang, Conghui Yin, Peng Xiao, Kelei Li, Meifang Tan

**Affiliations:** School of Automation, Wuhan University of Technology, 122 Luoshi Road, Wuhan 430070, Chinawfchang@whut.edu.cn (W.C.);

**Keywords:** traffic flow forecasting, attention mechanism, multi-input, spatial–temporal data

## Abstract

Accurate traffic flow forecasting is very important for urban planning and traffic management. However, this is a huge challenge due to the complex spatial–temporal relationships. Although the existing methods have researched spatial–temporal relationships, they neglect the long periodic aspects of traffic flow data, and thus cannot attain a satisfactory result. In this paper, we propose a novel model Attention-Based Spatial–Temporal Convolution Gated Recurrent Unit (ASTCG) to solve the traffic flow forecasting problem. ASTCG has two core components: the multi-input module and the STA-ConvGru module. Based on the cyclical nature of traffic flow data, the data input to the multi-input module are divided into three parts, near-neighbor data, daily-periodic data, and weekly-periodic data, thus enabling the model to better capture the time dependence. The STA-ConvGru module, formed by CNN, GRU, and attention mechanism, can capture both temporal and spatial dependencies of traffic flow. We evaluate our proposed model using real-world datasets and experiments show that the ASTCG model outperforms the state-of-the-art model.

## 1. Introduction

In the process of urbanization, traffic congestion poses an urgent issue that needs to be addressed. Many countries are implementing intelligent transportation systems [1], and real-time and accurate traffic flow prediction is a critical requirement for the establishment of such systems. With accurate traffic flow prediction, traffic management can anticipate future traffic conditions based on historical data, allowing people to plan their trips in advance and providing help for traffic guidance and route planning. However, traffic flow is influenced not only by the passage of time, but also by the interconnectedness of roads, forming a complex mesh structure [2,3]. Accurate traffic flow forecasting is a challenging task.

Fortunately, with the development of industry, many sensors [4] and other information-collecting devices are installed on traffic road networks. These devices can collect a large amount of data for research. Early methods based on statistical analysis, such as historical average (HA) [5], autoregressive integrated moving average (ARIMA) [6], Kalman filter (KF) [7], and exponential smoothing, can be used for traffic flow forecasting. However, they are limited in capturing the nonlinear dependence of time series and are unable to cope well with sudden changes in traffic flow. With the advancement of deep learning, deep learning models are used in many places, such as image processing, natural language processing, power prediction [8,9,10], etc. They have also gained attention in traffic flow prediction. Recurrent neural network (RNN) and its variants, such as long short-term memory (LSTM) [11], gated recurrent unit (GRU) [12], are common methods for time series prediction. While these models can handle nonlinear problems and perform well on single time series, they often overlook the spatial structure characteristics of the traffic road network and fail to utilize spatial correlation, resulting in suboptimal prediction performance. Some researchers have explored the use of convolutional neural networks (CNNs) [13] to model traffic flow data spatially, but CNNs struggle to capture temporal correlation, leading to limited results. To address both temporal and spatial correlation, many researchers have combined RNN and CNN to formulate integrated models for traffic flow prediction [14,15]. In recent years, attention mechanism has been proposed and applied to traffic flow forecasting [16,17,18], showing improved prediction accuracy compared to traditional methods, but there is still room for further improvement.

**Figure 1:**
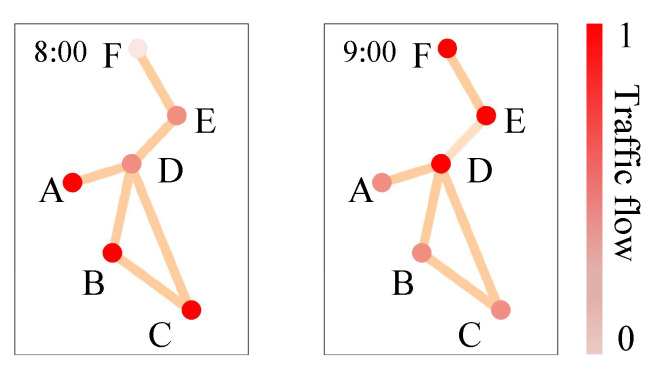
The spatial–temporal correlation of traffic flow.

Figure 1 shows the traffic flow network map at 8:00 and 9:00, respectively. The darker the color of the node, the higher the flow at that node. The traffic flow of nodes D, E, and F will be affected by nodes A, B, and C at previous moments. In other words, the traffic flow of each node is interrelated with other neighboring nodes [19]. When predicting the traffic flow of one node, the traffic flows of other nodes can also be properly input into the model. In addition, the traffic flow is also highly nonlinear and periodic [20], which makes it more difficult to predict. The traffic flow of the traffic road network is very dynamic in the temporal and spatial aspects, so it is a very challenging task to predict the traffic flow data accurately.

In order to address the above challenges, we propose the Attention-Based Spatial–Temporal Convolution Gated Recurrent Unit (ASTCG), which is employed to predict the traffic flow. This model combines CNN, GRU, and attention mechanism to accurately process traffic flow data. Our contributions of this paper are summarized as follows:

(1) Our proposed ASTCG model integrates CNN, GRU, and attention mechanism to capture both temporal and spatial correlations. GRU is utilized for capturing temporal correlation, while CNN is employed for capturing spatial correlation. ASTCG is also able to effectively utilize long history data due to the inclusion of the attention mechanism.

(2) Utilizing the periodic characteristics of traffic flow data, the data input is partitioned into three components in the model: near-neighbor data, daily-periodic data, and weekly-periodic data.

(3) By employing the real dataset PEMS for evaluation, we showcase that our proposed model outperforms the existing baseline models in terms of prediction accuracy.

The structure of this paper is as follows. Section 2 provides an overview of the research and development of traffic flow prediction. Section 3 presents the definition of the traffic flow prediction problem. In Section 4, we present the general framework and detailed architecture of our proposed model. Section 5 presents the experimental results of our model. Finally, Section 6 summarizes the entire paper.

## 2. Related Work

Statistical learning: Common statistical learning methods include KF, ARIMA, and Bayesian methods [21], which can be applied to traffic flow forecasting. The KF model assumes that the observed data are noisy and predicts future traffic flow based only on the state of the previous time step. However, KF is a linear prediction model and may have limitations in handling nonlinear and uncertain characteristics of traffic flow data. Shahriari et al. [22] combined bootstrap and ARIMA to improve the prediction accuracy while maintaining the ARIMA theory, but the prediction accuracy is poor when the flow changes suddenly. Thus, traditional statistical learning methods are limited by the assumption of stationary process and linear combinations, and may be less effective in predicting uncertain and complex traffic flow sequences, which may not meet the current practical engineering needs.

Machine learning: More scholars have studied how machine learning methods can be applied to the field of traffic flow forecasting than statistical learning methods. The traditional K-nearest neighbor (KNN) [23] and support vector machines [24] can model complex data, but they require detailed feature engineering and do not achieve the ideal results, so some scholars have improved them. Wang et al. [25] designed a KNN prediction algorithm with asymmetric loss and an asymmetric loss index, and the experimental results showed that when the asymmetric loss index decreased by more than 10%, the predicted value was closer to the upper edge of the actual traffic volume. Luo et al. [26] proposed a hybrid prediction method that combines discrete Fourier transform and support vector regression. The experimental results demonstrated that this algorithm achieves higher accuracy compared to traditional methods, making it an effective approach for holiday traffic flow prediction. Castro-Neto et al. [27] proposed an online supported support vector regression supervised statistical learning technique that can effectively and accurately predict short-term highway traffic flows for typical and atypical scenarios. Although machine learning methods are effective in capturing nonlinear features in traffic flow time series, they often require prior assumptions and extensive feature engineering to achieve excellent experimental results.

Deep learning: Since deep learning has powerful autonomous learning ability and nonlinear extraction capability, it has become an inevitable trend to apply deep learning in traffic flow forecasting problems [28,29,30,31]. The backpropagation neural network (BP) is one of the simplest neural network models. Chang et al. [32] utilized BP to forecast the traffic flow of a road section in Beijing during peak hours. RNN and its variants, LSTM and GRU [33], taking into account the correlation between multiple output data, so that the information at the previous time can be passed to the following cells, giving the neural network the function of memory, which is often used in the prediction of time series. CNN can extract spatial dependencies by convolutional operations, thus making full use of road network structure information for traffic flow forecasting [34,35]. Zheng et al. [36] combined CNN and LSTM to extract the spatial–temporal features of traffic flow. Zhai et al. [37] designed a novel self-supervised spatial–temporal holistic convolutional neural network to extract the temporal and spatial characteristics of traffic sequences, and the model has fewer parameters and faster inference speed. Since the spatial connection between multiple cross-sections in a traffic road network is an irregular data structure, the graph construction in GCN makes it more suitable for the representation of non-Euclidean spatial structure data, so some prediction methods construct a fixed graph structure based on the relationship on the actual geographic location of multiple cross-sections and construct prediction models on the fixed spatial structure graph to accomplish the task of multisection traffic flow [38]. Zhao et al. [39] designed a traffic speed prediction method based on temporal graphical convolutional networks, which unifies GCN and GRU in the special spatial–temporal component of the model, thus enabling the model to learn both non-Euclidean spatial features of the road network and temporal features of the traffic flow. Chang et al. [40] developed a novel framework called structure-learning convolution, which explicitly models structural information as convolution operations and thus designs local and global modules to learn static and dynamically changing structural information. Xu et al. [41] designed a novel hybrid adjacency matrix and combined it with a temporal attention mechanism for travel time prediction. Wang et al. [42] designed a trend space attention module whose main idea is to pass information between nodes with similar attributes to solve the spatial heterogeneity problem. Zhang et al. [43] extracted the spatial–temporal dependence of traffic flow by taking advantage of the graph attention mechanism for modeling non-Euclidean structured data and the LSTM cell for modeling time series. Guo et al. [44] introduced a latent network for spatial–temporal feature extraction in the prediction model to construct the dynamic road network graph adjacency matrix adaptively, and the experimental results showed that the adaptively learned dynamic Laplacian matrix has good ability to extract the spatial–temporal correlation of traffic data.

However, few scholars have considered how to make full use of the periodic features of traffic flow to improve the prediction accuracy, and even though Song et al. [45] used the periodic features of traffic flow data, its prediction effect is not made obvious by only adding the module of processing time inside the model. In the past studies, researchers have focused more on how to improve the internal structure of the model and ignored the influence of data input [46,47], but the prediction results are highly related to the input data of the model.

Motivated by the above research, we model traffic data using convolutional neural networks, gated recurrent units, attention mechanisms, and multiple input strategies considering the spatial–temporal dynamic correlation and periodicity of traffic flow data.

## 3. Preliminaries

The task of one-node traffic flow prediction involves predicting the number of vehicles passing through a specific section at future time intervals using historical traffic flow data from multiple sampling intervals. Since the one-node traffic flow is not only near-neighborly in the time dimension, but also exhibits the characteristics of daily and weekly cyclicity, as well as strong spatial correlations with neighboring sections, all of these nodes can impact the traffic flow values of the node in question at future time intervals. Therefore, incorporating traffic flow data from multiple neighboring nodes can lead to relatively accurate predictions of traffic flow at a particular node.

**Figure 2:**
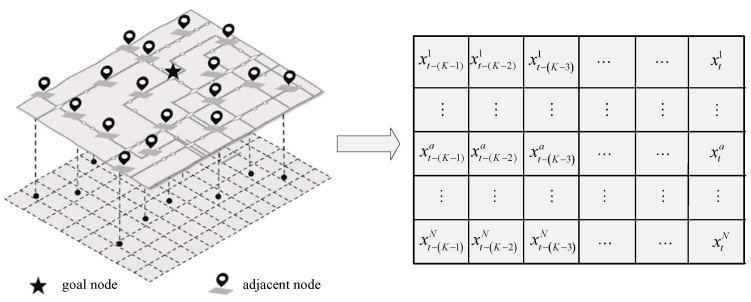
Spatial–temporal information structure of traffic flow.

As CNNs are commonly used for image data processing, feature extraction is achieved by scanning the gridded data in the image. Image data typically consist of multiple channels, with each channel containing small indivisible squares, each with its own unique location and pixel information. As illustrated in Figure 2, the spatial–temporal image of the traffic flow is constructed from the following three steps, based on the geographic distribution relationship between the goal node and its associated nodes.

(1) The plan is divided into a grid based on the relative position of each traffic sensor on the map, so that all the goal nodes and their associated nodes are divided into corresponding small squares, with each small square containing a traffic node.
(2) The traffic flow data recorded by the sensors at these nodes are filled in as pixel values in the small cells.
(3) This city sensor map is converted into a spatial–temporal image of traffic flow with length *N* squares and width *K* squares, where the coordinates of the goal node are (a,b), where 1<a<N,1<b<K, and the coordinates of the adjacent node are (n,k), where n=1,2,⋯,N;k=1,2,⋯,K;n≠a;k≠b.

Before introducing the one-node traffic flow prediction model, the input data, output data, and prediction tasks of the prediction model are mathematically defined. Assuming that there are *N* traffic nodes in the road network where the predicted goal node is located, the traffic flow collected by the sensor at the node of *n* can be defined as follows:

(1) $$X_{t}^{n}=\left\{x_{t-\left(K-1\right)}^{n},x_{t-\left(K-2\right)}^{n},\cdots ,x_{t}^{n}\right\}$$

where we define the traffic flow at time *t* and node *n* as $x_{t}^{n}$, n=1,2⋯,N. *K* represents the length of the input sequence.

In order to more accurately capture the dynamic correlation of traffic flows, three different temporal components, namely, near-neighbor data, daily-periodic data, and weekly-periodic data, denoted as $\left\{I^{r},I^{d},I^{w}\right\}$, are used as inputs to the model for feature extraction. Therefore, the historical traffic flow data of the goal node and its neighboring nodes form a spatial–temporal matrix $I^{r}$, which is mathematically defined as

(2) $$I^{r}=\left[\begin{array}{c} X_{t}^{1}\\ X_{t}^{2}\\ \vdots \\ X_{t}^{N} \end{array}\right]=\left[\begin{array}{cccc} x_{t-\left(K-1\right)}^{1} & x_{t-\left(K-2\right)}^{1} & \cdots & x_{t}^{1}\\ x_{t-\left(K-1\right)}^{2} & x_{t-\left(K-2\right)}^{2} & \cdots & x_{t}^{2}\\ \vdots & \vdots & \ddots & \vdots \\ x_{t-\left(K-1\right)}^{N} & x_{t-\left(K-2\right)}^{N} & \cdots & x_{t}^{N} \end{array}\right]$$

The travel patterns of people exhibit regularity, and traffic flow often show periodic fluctuations, such as morning and evening peaks on weekdays, that may exhibit similar traffic patterns. Additionally, traffic flow on weekdays may show similarities with the traffic flow of the previous weekday, and can be distinguished from nonworking days. Hence, in order to capture the daily and weekly cycle of the cross-sectional traffic flow data, two spatial–temporal matrices, $I^{d}$ and $I^{w}$, are constructed. The definitions of $I^{d}$ and $I^{w}$ are as follows:

(3) $$I^{d}=\left[\begin{array}{c} X_{t^{d}}^{1}\\ X_{t^{d}}^{2}\\ \vdots \\ X_{t^{d}}^{N} \end{array}\right]=\left[\begin{array}{cccc} x_{t^{d}-\left(K-1\right)}^{1} & x_{t^{d}-\left(K-2\right)}^{1} & \cdots & x_{t^{d}}^{1}\\ x_{t^{d}-\left(K-1\right)}^{2} & x_{t^{d}-\left(K-2\right)}^{2} & \cdots & x_{t^{d}}^{2}\\ \vdots & \vdots & \ddots & \vdots \\ x_{t^{d}-\left(K-1\right)}^{N} & x_{t^{d}-\left(K-2\right)}^{N} & \cdots & x_{t^{d}}^{N} \end{array}\right]$$

(4) $$I^{w}=\left[\begin{array}{c} X_{t^{w}}^{1}\\ X_{t^{w}}^{2}\\ \vdots \\ X_{t^{w}}^{N} \end{array}\right]=\left[\begin{array}{cccc} x_{t^{w}-\left(K-1\right)}^{1} & x_{t^{w}-\left(K-2\right)}^{1} & \cdots & x_{t^{w}}^{1}\\ x_{t^{w}-\left(K-1\right)}^{2} & x_{t^{w}-\left(K-2\right)}^{2} & \cdots & x_{t^{w}}^{2}\\ \vdots & \vdots & \ddots & \vdots \\ x_{t^{w}-\left(K-1\right)}^{N} & x_{t^{w}-\left(K-2\right)}^{N} & \cdots & x_{t^{w}}^{N} \end{array}\right]$$

where $t^{d}$ represents the moment at time *t* corresponding to the previous day, that is, $t^{d}=t-288$. $t^{w}$ represents the moment in the previous week that corresponds to time *t*, that is, $t^{w}=t-2016$. This is because in the traffic flow dataset, the time interval of recorded traffic flow data is 5 min, resulting in 288 traffic flow data points collected in one day, and 2016 traffic flow data points collected in one week.

The output of the one-node traffic flow is defined as follows:

(5) $$Out=X_{t}^{m}=\left\{x_{t+1}^{m},x_{t+2}^{m},\cdots ,x_{t+P}^{m}\right\}$$

where *m* represents the target cross-section in the road network, m=1,2,⋯,N; *x* represents the traffic flow of the node *m*.

Therefore, the one-node spatial–temporal traffic flow prediction task can be considered as learning a mapping function *F* from a large amount of traffic flow data $I=\left\{I^{r},I^{d},I^{w}\right\}$. Using this mapping function and the traffic flow data of the previous *K* moments, the traffic flow values of the future *P* moments are predicted, and its mathematical expression can be defined as follows:

(6) $$\left\{x_{t+1}^{m},x_{t+2}^{m},\cdots ,x_{t+P}^{m}\right\}=F\left(\left\{I^{r},I^{d},I^{w}\right\}\right)$$

## 4. Model Structure

**Figure 3:**
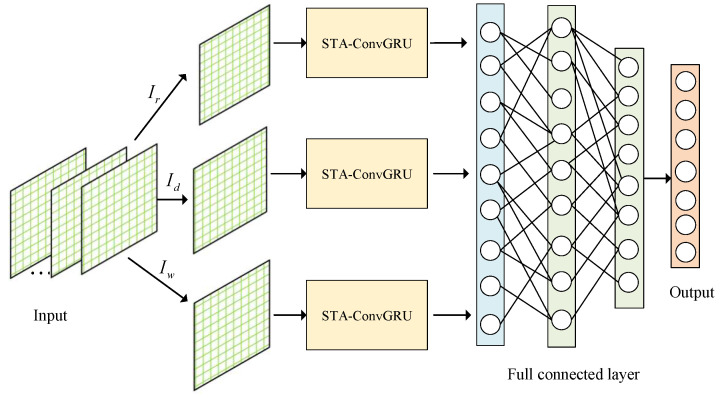
ASTCG architecture.

Figure 3 illustrates the overall structure of the ASTCG model. The STA-ConvGRU module integrates the fine-grained feature extraction capability of CNN, the efficient temporal relationship modeling of GRU, and the attention mechanism for focus capturing. The input sequence is that the spatial information of the goal node is processed through CNN convolutional and pooling layers, which is passed to GRU for further processing. The attention module quantifies the historical information in the traffic flow sequence, addressing the limitation of GRU in distinguishing important and unimportant information in the sequence. Subsequently, near-neighbor data, daily-periodic data, and weekly-periodic data are used as input to the STA-ConvGRU module, enabling finer-grained extraction of spatial–temporal characteristics of the traffic flow at the goal node and reducing the random influence of uncertainty on the overall traffic flow distribution. Finally, the outputs of the three components are concatenated and transformed into feature vector data, and the prediction results are obtained through two fully connected layers.

### 4.1. ConvGRU Module

**Figure 4:**
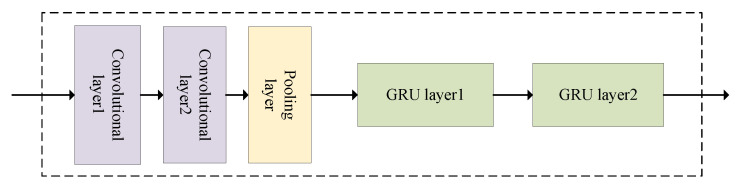
ConvGRU module structure.

The ConvGRU module consists of CNN and GRU, where the convolutional kernel sliding operation of CNN captures the spatial correlation of traffic flow at a fine granularity, and the special gating unit mechanism in GRU efficiently extracts the temporal dependence of traffic flow. The combination of CNN and GRU in the ConvGRU module is shown in Figure 4. The CNN contains two convolutional layers and one pooling layer, which is due to the complex spatial characteristics of the traffic road network and the limited expression capability of the single-layer convolutional kernel, so two layers of convolutional layer 1 and convolutional layer 2 are used to extract more comprehensive spatial correlation, followed by filtering unnecessary information as well as reducing the dimensionality of the input data through the pooling layer; finally, the output of the CNN pooling layer is used as the input of GRU, and the output value of the module is obtained after two layers of GRU.

The input of the ConvGRU module is represented as $I={\left[X_{t}^{1},X_{t}^{2},\cdots ,X_{t}^{N}\right]}^{T}$. In the convolution layer 1 and convolution layer 2, a 1D convolution operation is selected to process the input spatial–temporal traffic flow data, and the spatial influence of adjacent nodes on the traffic flow of the goal node is extracted by sliding the convolution kernel over the input data. The convolution layer 1 and convolution layer 2 are calculated as follows:

(7) $$Y_{1}=\sigma \left(W_{c1}*I+b_{c1}\right)$$

(8) $$Y_{2}=\sigma \left(W_{c2}*Y_{1}+b_{c2}\right)$$

where $W_{c1},W_{c2}$ are the weight parameters of the convolution kernel; $b_{c1},b_{c2}$ are the deviation parameters of the convolution kernel; ∗ represents the convolution operation; σ(·) is the activation function; $Y_{1},Y_{2}$ are the outputs of convolution layer 1 and convolution layer 2.

Pooling layers are useful to speed up the computation and prevent overfitting. This is because the pooling layer can effectively reduce the size of the parameter matrix, thus reducing the number of parameters in the final connection layer. During the pooling process, a large amount of useless data are filtered out, thus ensuring better extraction capability of the model when processing traffic flow data. After the pooling layer is processed, the multidimensional data are converted to a 1D sequence by using the flatten() operation.

GRU is an improved model based on RNN, which is a type of self-mapping neural network with strong computational power and long-term memory. GRU has two gating structures, namely, the update gate $z_{t}$ and the reset gate $r_{t}$. The update gate determines how much information from the previous time step is incorporated to update the information of the unit at the current time step. On the other hand, the reset gate determines the degree of ignoring information from the previous time step. The calculation formulas for $z_{t}$ and $r_{t}$ are as follows:

(9) $$z_{t}=\sigma \left(w_{z}*\left[h_{t-1},x_{t}\right]+b_{z}\right)$$

(10) $$r_{t}=\sigma \left(w_{r}*\left[h_{t-1},x_{t}\right]+b_{r}\right)$$

where $w_{z},w_{r}$ are the weight matrices of the update gate and reset gate; $x_{t}$ is the input of the current cell, and $h_{t-1}$ is the state information of the cell at the previous moment.

The cell state information at each time step in GRU is passed on to the next time step. $h_{t}$ represents the output value of the cell at the current time step, and its expression is as follows:

(11) $$h_{t}=\left(1-z_{t}\right)*h_{t-1}+z_{t}*h_{t}^{\prime }$$

(12) $$h_{t}^{\prime }=\tanh\left(w_{h}*\left[r_{t}*h_{t-1}\right],x_{t}\right)$$

Compared to traditional RNN, GRU is capable of better learning time-dependent features due to the presence of update and reset gates, which mitigate the issues of gradient explosion and gradient disappearance that may arise when dealing with long sequences of data. In this model, two layers of GRU are stacked to extract the time-dependent features of traffic flow. The GRU units take the temporal data input for each time window and the hidden state as input.

In our model, the output $C_{out}=\left[C_{t-(K-1)},C_{t-(K-2)},\cdots ,C_{t}\right]$ of the CNN serves as the input for the first layer of GRU units, and the hidden state value of the first layer of GRU units is used as the input for the subsequent layer of GRU. The formula for each layer of GRU units is as follows:

(13) $$Z_{t}=\sigma \left(W_{z}*\left[H_{t-1},F_{t}\right]+b_{z}\right)$$

(14) $$R_{t}=\sigma \left(W_{r}*\left[H_{t-1},F_{t}\right]+b_{r}\right)$$

(15) $$H_{t}^{\prime }=\tanh\left(W_{h}*\left[R_{t}*H_{t-1}\right],F_{t}\right)$$

(16) $$H_{t}=\left(1-Z_{t}\right)*H_{t-1}+Z_{t}*H_{t}^{\prime }$$

where $F_{t}$ represents the input of the GRU at time *t*; σ(·) and tanh(·) are the activation function; $W_{z},W_{h},W_{r}$ are the weight parameters; $b_{z},b_{r}$ are the deviation parameters; $Z_{t},R_{t}$ represent the output of the update gate and the reset gate; $H_{t}$ is the output of the GRU unit.

### 4.2. STA-ConvGRU Module

**Figure 5:**
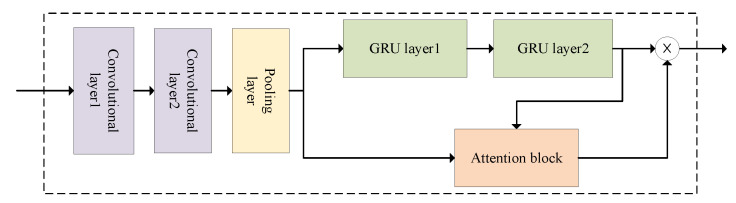
STA-ConvGRU module structure.

The historical traffic flow information at different moments has varying effects on the prediction results. However, the GRU is unable to identify the key sequence information in the traffic flow sequence, leading to all the information in the input sequence being equally calculated. This can result in decreased model prediction accuracy and increased computation time. To address this issue, we designed the STA-ConvGRU module, which incorporates the attention mechanism to reduce attention to unimportant information. This allows us to obtain potential information during traffic flow changes and quantify the importance of historical traffic flow data at different locations and moments. Figure 5 depicts the structure of the STA-ConvGRU module, where the outputs of the CNN and the ConvGRU module are combined as input to the temporal attention mechanism module. The attention coefficients calculated by the module are then combined with the output of the ConvGRU module to obtain the final output of the STA-ConvGRU module.

In the attention module, each element of the input traffic flow sequence is assigned a corresponding attention allocation probability, which is calculated internally as

(17) $$S_{t}=W_{a3}\tanh\left(W_{a1}*C_{out}+W_{a2}*H_{2,t}\right)$$

where $W_{a3},W_{a2},W_{a1}$ are the weight parameters; $C_{out}$ is the output of the CNN; $S_{t}=\left(s_{t-(K-1)},s_{t-(K-2)},\cdots ,s_{t}\right)$ represents the importance of each historical moment of the traffic flow sequence.

The attention coefficient is defined as

(18) $$a_{t-n}=\frac{\exp\left(s_{t-n}\right)}{\sum_{n=0}^{n=K}\exp(s_{t-n})}$$

where $a_{t-n}$ is the attention factor, which represents the degree of influence of each historical traffic flow time step on future traffic flow, n=0,1,⋯,K. Therefore, the output of the ConvGRU module at each time step is multiplied by the attention coefficient and summed to obtain the output of the STA-ConvGRU module. The calculation formula for the output of the STA-ConvGRU module is as follows:

(19) $$H_{a,t}=\sum_{n=0}^{n=K}a_{t-n}h_{2,t-n}$$

## 5. Experiments

### 5.1. Experimental Setup

#### 5.1.1. Experimental Data

The datasets used in this paper are PEMS04 and PEMS08, which are real-time traffic flow datasets collected by Caltrans Performance Measurement System. The data are collected every 30 s and then aggregated every 5 min. A brief description of these datasets is listed in Table 1.

The PEMS04 dataset consists of feature data in three dimensions: traffic flow, average speed, and average lane occupancy. It includes data from 307 nodes in the San Francisco Bay Area and spans from 1 January 2018 to 28 February 2018.

The PEMS08 dataset comprises feature data for 170 nodes in Los Angeles County, including traffic flow, average speed, and average lane occupancy. The dataset spans from 1 July 2016 to 31 August 2016.

In the experimental part, nodes 104 and 307 are chosen as the goal nodes from the PEMS04 dataset. The dataset is divided into 6:2:2, with 35 days (1 January 2018 to 4 February 2018) for the training set, 12 days (5 February 2018 to 16 February 2018) for the validation set, and 12 days (17 February 2018 to 28 February 2018) for the test set. For the PEMS08 dataset, nodes 58 and 100 are selected as the goal nodes. The dataset is divided into 38 days (1 July 2016 to 7 August 2016) for the training set, 12 days (8 August 2016 to 19 August 2016) for the validation set, and 12 days (20 August 2016 to 31 August 2016) for the test set.

Data normalization is necessary due to the significant variability of traffic flow data at different moments, which can influence model training and testing.

(20) $$X=\frac{X-X_{\min}}{X_{\max}-X_{\min}}$$

where $X_{\max},X_{\min}$ represent the maximum and minimum values in the traffic flow sequence.

#### 5.1.2. Hyperparameter Settings

Our ASTCG model is implemented using TensorFlow 1.14 and is run on an Nvidia GeForce RTX 2080Ti GPU. In the experiments, the convolution layer is configured with 15 convolutional kernel channels, each with a size of 7. The sliding window step size for input traffic flow data is set to 1, and computational padding is applied based on the size of the convolution kernels to ensure that the convolution output size matches the input size. The GRU is configured with 24 output units, fully connected layer 1 and fully connected layer 2 have 20 and 10 output units, respectively, and the output layer has 12 output units. During model training, the model is trained in 70 batches with a data batch size of 128, using the Adam optimizer for optimization. The Adam algorithm is an effective stochastic optimization algorithm that combines first-order moment estimation of the gradient and second-order moment estimates to update the parameters. The time interval of the dataset is 5 min, and the historical time length *K* is set to 12 (representing one hour in the past), while the prediction length *P* is set to 12 (representing one hour in the future).

#### 5.1.3. Evaluation Metrics

For all prediction models, we use mean absolute error (MAE), root mean square error (RMSE), and mean absolute percentage error (MAPE) as our evaluation metrics to assess the performance of the model, The three are calculated as follows.

Mean Absolute Error (MAE):

(21) $$\mathrm{MAE}=\frac{1}{N}\left(\sum_{i=1}^{N}\left|\left(y_{i}-{\hat{y}}_{i}\right)\right|\right)$$

Root Mean Square Error (RMSE):

(22) $$\mathrm{RMSE}=\sqrt{\frac{1}{N}\left({\sum_{i=1}^{N}\left(y_{i}-{\hat{y}}_{i}\right)}^{2}\right)}$$

Mean Absolute Percentage Error (MAPE):

(23) $$\mathrm{MAPE}=\frac{100\%}{N}\sum_{i=1}^{N}\left|\frac{y_{i}-{\hat{y}}_{i}}{y_{i}}\right|$$

#### 5.1.4. Compared Methods

In the experimental part, HA, BP, GRU, LSTM, and Spatial–Temporal Graph Convolution Network (STGCN) and Temporal Graph Convolutional Network (T-GCN), are used as the baseline models. The dataset division and hyperparameter settings for the baseline models are kept consistent with the proposed model to ensure fair evaluation of their performance. The details of the baseline models are shown below:

HA [5]: The HA model treats the traffic flow sequence as a seasonal process and generates predictions by taking the weighted average of previous seasons.

BP [32]: BP is a multilayer feedforward network trained using the error backpropagation algorithm. It captures the nonlinear mapping relationship within the traffic flow sequence and dynamically adjusts the weights and thresholds of the network through backpropagation, ensuring that the predicted values are consistently close to the true values.

LSTM [11]: Similar to the GRU, the LSTM also utilizes internal gating units to control the flow of historical information, enabling effective management of historical traffic flow data and achieving high prediction performance.

GRU [12]: The gating unit in GRU effectively captures the time dependence of traffic flow while addressing the issues of gradient explosion and gradient disappearance that can arise from long sequences.

STGCN [48]: It utilizes the ChebNet and 2D convolutional network to model spatial–temporal graph data, offering fast training speed and low model complexity.

T-GCN [39]: T-GCN is a spatial–temporal data mining model that leverages the combination of GCN and GRU to extract spatial–temporal features for accurate traffic flow prediction.

### 5.2. Experimental Results

Table 2 presents the comparison of our proposed method with other baselines on PEMS04 dataset, while Table 3 displays the comparison of our proposed method with other baselines on PEMS08 dataset. The results clearly demonstrate that our proposed ASTCG model surpasses all baseline models in terms of all evaluation metrics.

The prediction results of traditional time series forecasting methods are not satisfactory, indicating their limited ability in dealing with complex spatial–temporal traffic flow data. The poor performance of the HA model can be attributed to its simplistic approach of taking the average value of traffic flow data from previous moments as the prediction for the next moment, without considering the nonlinear temporal variations in traffic flow. While the BP model accounts for the nonlinearity and instability of traffic flow, it lacks consideration for the time dependence of traffic flow. This limitation results in inferior prediction performance compared to GRU and LSTM models, which have gating units that effectively capture both short-term and long-term dependencies in time series data. The STGCN and T-GCN models not only incorporate the temporal dependence of traffic flow, but also incorporate a spatial extraction component to capture spatial features of all nodes. This design effectively enhances the prediction performance of the models, surpassing the prediction models LSTM and GRU, which only consider temporal features. The MAE, RMSE, and MAPE of the STGCN model are 4.13%, 2.86%, and 2.98% lower than that of the GRU model at node 307 of the PEMS04 dataset. The MAE, RMSE, and MAPE of the STGCN model are decreased by 6.89%, 5.81%, and 6.18% compared to the GRU model at node 100 of the PEMS08 dataset.

Our proposed ASTCG model addresses long time dependencies and complex spatial structures by combining GRU, CNN, and self-attention mechanism. The data input is divided into three parts, including near-neighbor data, daily-periodic data, and weekly-periodic data, which are incorporated into the model. The ASTCG model achieved the best prediction results among all baseline models, with MAE values reduced by 10.08% relative to the T-GCN model, and RMSE values reduced by 9.83% relative to the STGCN model on the prediction task at node 307 of the PEMS04 dataset. On the prediction task at node 100 of the PEMS08 dataset, the MAE value is reduced by 7.60% and the RMSE value is reduced by 3.40% relative to the T-GCN model, which indicates that the ASTCG model can effectively enhance the extraction of spatial–temporal features of traffic flow.

**Figure 6:**
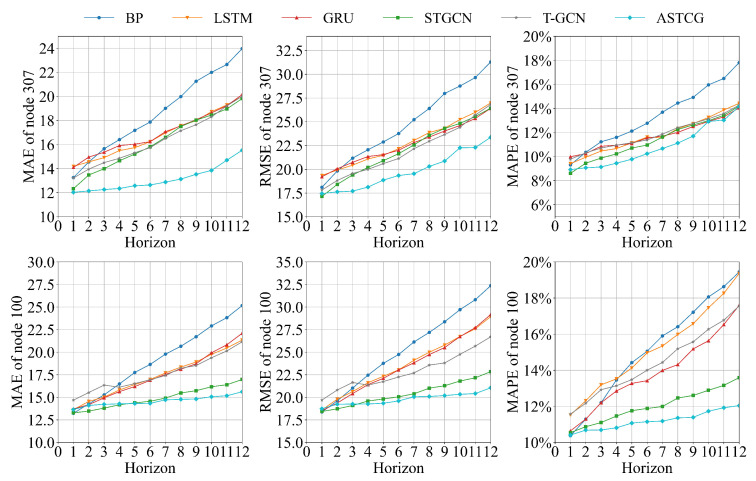
Evaluation metrics of different models on the PEMS04 dataset.

**Figure 7:**
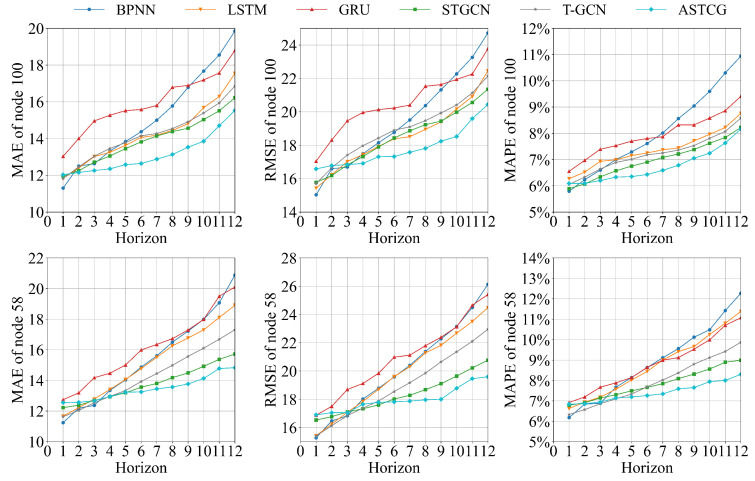
Evaluation metrics of different models on the PEMS08 dataset.

Figure 6 and Figure 7 present the MAE, RMSE, and MAPE evaluation metric values for different models on the PEMS04 dataset and the PEMS08 dataset for various time-step prediction tasks. The ASTCG model consistently achieves the best prediction performance across all prediction time steps, as indicated by the lower values of the evaluation metrics. This demonstrates the advantage of the ASTCG model in long-term traffic flow prediction. It is observed that the prediction performance of all baseline models deteriorates with increasing prediction intervals, which is expected as longer prediction time steps provide less useful data for the prediction model to learn from. The ASTCG model exhibits a similar decay rate compared to the STGCN and T-GCN models in the first four time steps. However, as the time step increases, the decay rate of the ASTCG model is lower than the other two models, indicating its superior ability in extracting temporal features. This implies that the ASTCG model can still extract useful information from historical data even as the prediction time step increases, highlighting the effectiveness of the attention mechanism in quantifying temporal correlations in traffic flow sequences.

**Figure 8:**
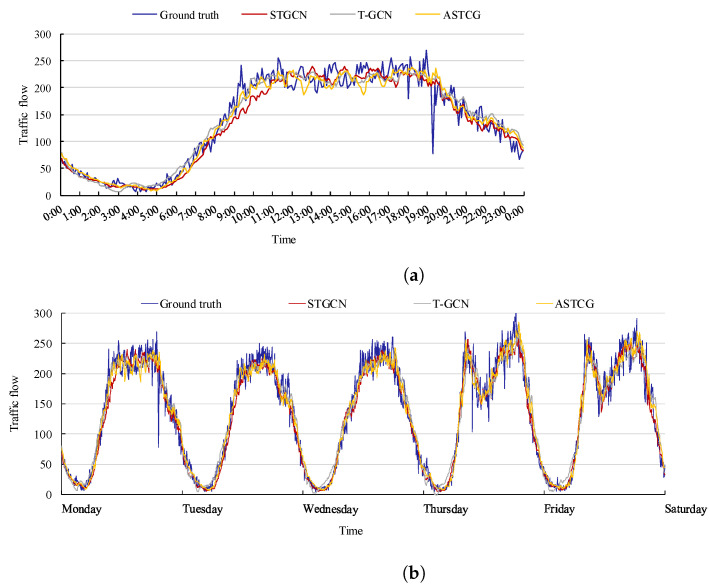
Traffic flow visualization of three spatial–temporal prediction models on the PEMS04 dataset. (**a**) One-day traffic flow visualization at node 104; (**b**) One-week traffic flow visualization at node 104.

**Figure 9:**
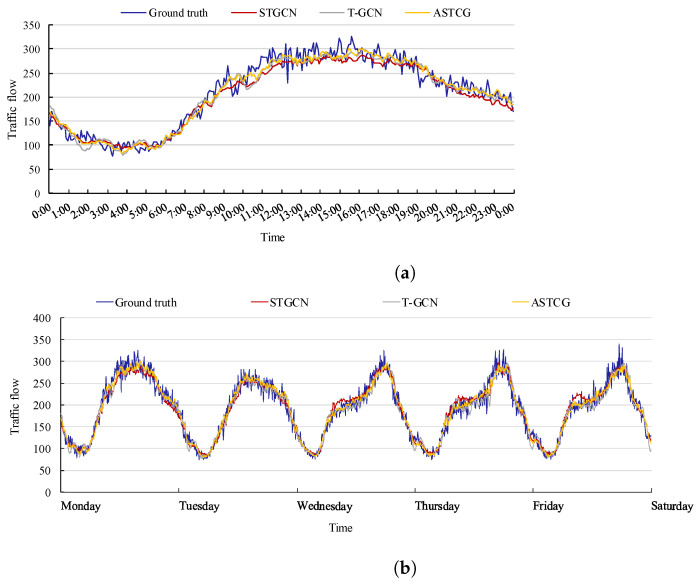
Traffic flow visualization of three spatial–temporal prediction models on the PEMS08 dataset. (**a**) One-day traffic flow visualization at node 58; (**b**) One-week traffic flow visualization at node 58.

To effectively showcase the prediction performance of the ASTCG model, we visualize the traffic flows of STGCN, T-GCN, and ASTCG for one day and one week at node 104 on the PEMS04 dataset and node 58 on the PEMS08 dataset. Figure 8 and Figure 9 depict that the real traffic flow is more accurately followed and the prediction accuracy is higher with the ASTCG model compared to the STGCN and T-GCN.

### 5.3. Ablation Experiments

The ASTCG model comprises three main components, including a convolutional recurrent network component, an attention mechanism, and multiple input modules for near-neighbor data, daily-periodic data, and weekly-periodic data. The model is evaluated for traffic flow prediction tasks at various time intervals, such as 15 min, 30 min, and 60 min, at node 307 of the PEMS04 dataset, and node 100 of the PEMS08 dataset. The experimental results are presented in Table 4, and the specific details of the three variants of the model are as follows:

(a) ConvGRU: This model seamlessly combines a convolutional neural network with a recurrent neural network to capture the spatial–temporal dependencies of traffic flow data.
(b) STA-ConvGRU: This model enhances ConvGRU by incorporating an attention module that quantifies the importance of historical time steps for improved prediction accuracy.
(c) MI-ConvGRU: This model extends ConvGRU by incorporating a multi-input component for temporal data, which captures temporal dependence of traffic flow from multiple aspects by incorporating near-neighbor data, daily-periodic data, and weekly-periodic data as inputs.

The experimental results show that the prediction performance of all four models declines as the length of the prediction time step increases on both datasets. This is mainly attributed to the reduced knowledge that the models can glean from historical traffic flow data when predicting larger time steps, resulting in decreased prediction accuracy. Additionally, it can be observed that the STA-ConvGRU and MI-ConvGRU models outperform the ConvGRU model in terms of prediction performance. This is reasonable, as the STA-ConvGRU and MI-ConvGRU models incorporate different temporal dependency extraction components, which enable them to capture richer time-related information. This finding further validates the effectiveness of the proposed multi-input component and attention mechanism component. Among the four models, the ASTCG model exhibits the best prediction performance. For instance, in the 60 min prediction task at node 100 of the PEMS08 dataset, the MAE value of ASTCG is reduced by 2.72% compared to MI-ConvGRU, and the MAPE value is reduced by 2.80% compared to STA-ConvGRU. This indicates that the inclusion of the feature extraction component in the model effectively enhances the prediction accuracy. Moreover, the ASTCG model demonstrates accurate predictions of traffic flow at four different nodes in the two datasets, showcasing its excellent generalization ability.

**Figure 10:**
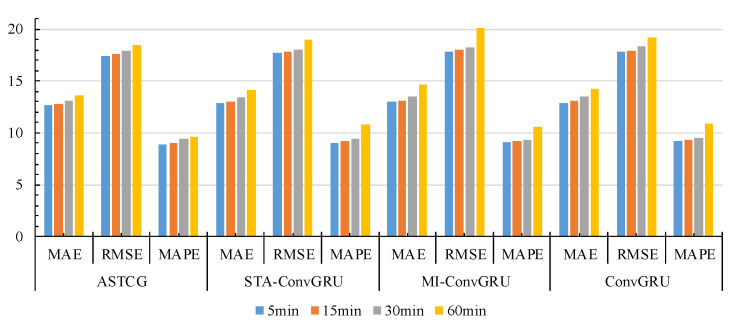
Prediction performance of the four models at node 307 of the PEMS04 dataset.

**Figure 11:**
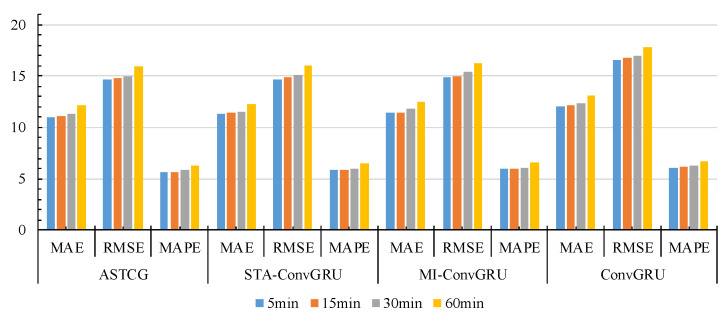
Prediction performance of the four models at node 100 of the PEMS08 dataset.

To visually compare the prediction performance of the four models, Figure 10 and Figure 11 display the prediction evaluation metrics at node 307 of the PEMS04 dataset and node 100 of the PEMS08 dataset. It is evident from the visualizations that ASTCG consistently achieves the best prediction results for all four evaluation metrics at different prediction time steps, showcasing the superior performance of this model.

## 6. Conclusions

In this paper, we propose a new spatial–temporal attention-based model, Attention-Based Spatial–Temporal Convolution Gated Recurrent Unit (ASTCG), applied to traffic flow forecasting. The model combines a convolution neural network, a gated recurrent unit, and a spatial–temporal attention mechanism to capture the spatial–temporal correlation of traffic flow. Furthermore, our model leverages the cyclic nature of traffic flow data by incorporating near-neighbor data, daily-periodic data, and weekly-periodic data, which enhances the prediction accuracy. Our proposed model is tested on two real datasets and outperforms all baseline methods. However, it should be noted that traffic flow prediction is influenced by various factors, such as weather, holidays, and social events. In future research, it would be beneficial to consider these factors to further improve the effectiveness of the model. We want the research work in this paper to reach cooperation with related enterprises or traffic management departments so that it can provide data support for route planning and traffic guidance, and help people to travel on a daily basis.

## Figures and Tables

**Table 1:** Datasets description.

Datasets	PEMS04	PEMS08
Goal node number	104, 307	58, 100
Number of node	307	170
Train time range	1 January 2018–4 February 2018	1 July 2016–7 August 2016
Validation time range	5 February 2018–16 February 2018	8 August 2016–19 August 2016
Test time range	17 February 2018–28 February 2018	20 August 2016–31 August 2016

**Table 2:** Comparison with different baselines on PEMS04.

Model	Node 307 of the PEMS04			Node 104 of the PEMS04		
	MAE	RMSE	MAPE	MAE	RMSE	MAPE
HA	19.73	26.32	18.04%	20.32	26.18	18.41%
BP	18.64	25.07	13.39%	19.14	25.73	15.23%
LSTM	16.81	22.94	11.78%	17.40	23.87	15.21%
GRU	16.94	22.72	11.76%	17.39	23.75	13.91%
STGCN	16.24	22.07	11.41%	14.94	20.43	12.02%
T-GCN	16.36	21.96	11.34%	16.48	22.45	12.50%
ASTCG	14.71	19.90	10.84%	14.58	19.80	11.20%

**Table 3:** Comparison with different baselines on PEMS08.

Model	Node 100 of the PEMS08			Node 58 of the PEMS08		
	MAE	RMSE	MAPE	MAE	RMSE	MAPE
HA	16.7	21.45	8.45%	16.21	21.32	9.12%
BP	15.13	19.71	8.08%	15.44	20.48	8.94%
LSTM	14.26	18.66	7.37%	15.14	20.10	8.77%
GRU	14.95	19.62	7.44%	15.12	20.11	8.81%
STGCN	13.92	18.48	6.98%	13.78	18.33	7.83%
T-GCN	14.21	18.93	7.22%	14.29	19.02	7.95%
ASTCG	13.13	17.85	6.47%	13.47	18.01	7.42%

**Table 4:** Evaluation metric values of ASTCG and three variants of the model at different time steps.

Model	Horizon	Node 307 of the PEMS04			Node 100 of the PEMS08		
		MAE	RMSE	MAPE	MAE	RMSE	MAPE
ConvGRU	5 min	12.89	17.81	9.23%	12.02	16.57	6.09%
	15 min	13.12	17.91	9.36%	12.14	16.75	6.12%
	30 min	13.46	18.33	9.48%	12.33	16.95	6.25%
	60 min	14.22	19.18	10.84%	13.13	17.86	6.74%
STA-ConvGRU	5 min	12.91	17.68	8.99%	11.28	14.71	5.87%
	15 min	12.97	17.78	9.21%	11.42	14.92	5.89%
	30 min	13.36	18.02	9.43%	11.55	15.12	5.98%
	60 min	14.11	19.01	10.8%	12.28	16.01	6.43%
MI-ConvGRU	5 min	12.98	17.77	9.09%	11.38	14.86	5.92%
	15 min	13.14	18.02	9.21%	11.45	14.98	5.95%
	30 min	13.54	18.23	9.35%	11.80	15.43	6.11%
	60 min	14.69	20.10	10.54%	12.47	16.23	6.55%
ASTCG	5 min	12.72	17.44	8.91%	11.03	14.71	5.63%
	15 min	12.82	17.58	9.04%	11.09	14.75	5.68%
	30 min	13.12	17.91	9.46%	11.31	14.98	5.85%
	60 min	13.59	18.47	9.66%	12.13	15.89	6.25%

## Data Availability

A publicly available dataset was analyzed in this study. It can be found here: https://github.com/wanhuaiyu/ASTGCN/tree/master/data (accessed on 1 May 2023).
